# “Deadly”, “fierce”, “shameful”: notions of antiretroviral therapy, stigma and masculinities intersecting men’s life-course in Blantyre, Malawi

**DOI:** 10.1186/s12889-021-12314-2

**Published:** 2021-12-11

**Authors:** Astrid Berner-Rodoreda, Esther Ngwira, Yussif Alhassan, Boniface Chione, Rosalia Dambe, Till Bärnighausen, Sam Phiri, Miriam Taegtmeyer, Florian Neuhann

**Affiliations:** 1 Heidelberg Institute of Global Health, University of Heidelberg, Heidelberg, Germany; 2 Lilongwe University of Agriculture and Natural Resources, Lilongwe, Malawi; 3 Community Health Systems Group, Department of International Public Health, Liverpool School of Tropical Medicine, Liverpool, UK; 4 The Lighthouse Trust, Blantyre, Malawi

**Keywords:** Stigma, Men having sex with women, Masculinities, HIV, ART, Malawi, Qualitative, Interviews

## Abstract

**Background:**

Stigma and masculinity represent persistent barriers in delivering successful HIV interventions to men. Our study examined community perceptions of HIV and anti-retroviral therapy (ART) and their implications for men on ART across the life course in Blantyre, Malawi.

**Methods:**

Our qualitative study is based on 72 face-to-face semi-structured interviews. Participants were selected purposively and included men on ART (with suppressed and unsuppressed viral loads), adult male community members irrespective of HIV status and other HIV stakeholders such as health personnel and program implementers. Interviews were conducted in Chichewa and English, transcribed verbatim and analyzed thematically in NVivo 12. We applied the socio-ecological model as our theoretical framework as well as a “life-course” perspective.

**Results:**

Our findings highlight lingering negative perceptions towards ART in general and towards PLHIV irrespective of viral load suppression. With intersecting notions of masculinity and stigma, men’s descriptions of anticipated stigma in their relationships and when visiting health facilities dominated. Stigma was experienced at the personal, interpersonal, facility and community level. Yet, men living with HIV were perceived differently throughout the life-course, with young sexually active men seen as the most stigmatized group and older men seen as drawing resilience from a greater range of masculine norms. Some men of all ages displayed “transformative” masculinities independent of stigma and community expectations.

**Conclusions:**

We propose the “life-course” as a useful concept for studies on masculinity, HIV and stigma. Considering gendered constructions of “respectable” midlife-older age vis-à-vis younger age, and how they influence stigma as well as uptake and adherence to ART might lead to more targeted services for men that build on “transformative masculinities”.

**Supplementary Information:**

The online version contains supplementary material available at 10.1186/s12889-021-12314-2.

## Introduction

Stigma and discrimination of people living with HIV (PLHIV) are persistent barriers to holistic HIV care [1–5]. A recent bibliometric analysis [6] showed stigma issues are still relevant despite decades of HIV interventions and a plethora of HIV-related stigma conceptualizations. Herek distinguished between symbolic stigma (based on assumptions towards affected groups such as homosexuals), instrumental stigma (beliefs about transmission risks from infected individuals) and general stigma towards PLHIV [7]. Two studies from South Africa expand on the impacts of perceived stigma (attitudes and behavior PLHIV believe others may display towards them) and enacted stigma (used synonymously with discrimination) [8, 9]. Social psychologists have further added a general framework with public stigma at the center of structural stigma (stigma inflicted through institutions, laws and social context), stigma by association (stigma towards people close to a stigmatized person) and self-stigma (defined as anticipated, experienced or internalized stigma) [10]. In this article we focus on the forms of stigma most spoken about by our participants: communal or general stigma, anticipated, enacted and, to a lesser extent, self-stigma.

HIV-related stigma is profoundly gendered in nature [11] with discourses on masculinities and stigma intersecting. Men who have sex with women (MSW) have long been absent from HIV policies and interventions, often being seen as “transmitters” in relation to HIV prevention in women [12, 13]. MSW were initially not recognized as an important target group in their own right despite late presentation for treatment and higher mortality [14, 15] when compared to women [16–18]. Masculinity and stigma research in this group has highlighted structural barriers to HIV-testing [19]; delaying testing until sick [20]; a preference for private settings [21], and adherence problems [22] including missed appointments [23]. Further, the intersection of stigma and masculinity norms create barriers to men’s utilization of HIV services [24–26].

In our study, we draw on Connell’s concept of “hegemonic masculinity” to understand men’s heath seeking behavior. Connell adapted Gramsci’s use of “hegemony” or dominance in terms of internalized power relations to gender which “guarantees (or is taken to guarantee) the dominant position of men and the subordination of women” [27]. “Hegemonic masculinity” was normatively configured as “the most honored way of being a man” [28] and moved the masculinity discourse from a “single normative male sex role” [29] to multiple masculinities, emphasizing relationality and a hierarchical order of masculinities which could change with context.

Our study explores differing community perceptions of HIV, ART, and community views of male PLHIV of different ages. It further explores MSWs’ behavior and engagement with HIV and ART services with a view to understanding the intersection of HIV-related stigma and masculinities in the community contexts these play out in. As men engage as individuals, in relationships, in relation to facilities and as community members we will summarize our findings by using the socio-ecological model (SEM), which emphasizes the centrality of the intrapersonal, interpersonal and societal context in shaping human behavior [30]. Based on this model, stigma may be conceptualized as “constructed” and “experienced in different dimensions of men’s lives. While the life-span was originally part of SEM [31], it got lost in some adaptations to other disciplines [32].

## Methods

We conducted the study in urban and surrounding semi-urban and rural settlements of Blantyre district – the Malawian district with the highest HIV prevalence (17.7%) and lowest viral load suppression rates (59.5%) (Ministry of Health, Malawi, 2018). Blantyre district provides primary and secondary health services with Queen Elizabeth Central Hospital as the largest tertiary referral and teaching hospital in the Southern region of Malawi. We included Umodzi Family Centre (UFC), a Lighthouse Trust operated ART facility affiliated to the Central Hospital, in our sampling frame. In 2020 UFC was providing comprehensive integrated HIV care services to over 14,000 clients (42% of them male), about 15% of all patients on ART in Blantyre [33].

### Study design, sampling, data collection and analysis

We used qualitative methods to explore perceptions and personal experiences of ART and HIV-related services among men and chose in-depth interviews to allow men and other stakeholders to speak freely without peer pressure at a time and place convenient to them. We nested our work within a “Men on ART” study conducted by the Lighthouse Trust and the University of Heidelberg on viral load suppression among MSW in Blantyre and conducted interviews in November/December 2019 after establishing prior contact with main stakeholders in August and September 2019. Observations, mostly at UFC, enhanced the interview-based data. We purposively sampled men living with HIV receiving ART from UFC laboratory lists based on being over 18 years, having sufficient contact details and a viral load (VL) result within the last 6 months. We included those with suppressed (defined as <1000 copies/ml) and unsuppressed viral loads (defined as >1000 copies/ml) [34]. Potential interviewees were informed that the research team were interested in hearing from men with suppressed and unsuppressed VLs to better understand male issues regarding viral load suppression.

 Male community participants were interviewed to gain an outsider perspective; they were sampled from different age groups and communities of stratified wealth: one rural community, two semi-urban communities and an urban youth center in Blantyre city. Finally, we purposively sampled district stakeholders including health personnel, some of whom served as board members of the District Health Office, HIV program implementers and HIV researchers with experience of working with male PLHIV. All interviewees consented to the interview.

Interviews were conducted in Chichewa or English by an experienced mixed gender research team which included the lead and 2nd author. The lead author, a female trained social anthropologist and PhD candidate with many years’ experience of working with different stakeholders and communities in Sub-Saharan Africa, developed a training schedule for the two research assistants (one man and one woman with prior experience of qualitative research) covering qualitative methods, ethical considerations^1^, background on the study and the piloting of the interview guides. We conducted audio-recorded interviews with stakeholders at their workplaces; with men on ART at the health facility; with men in the communities at home or a convenient, quiet place (church premises or headman’s compound) and at a youth center. Interview guides covered community perceptions of HIV and ART; experience of (HIV-related) health services and of ART. They contained no direct questions on stigma. Interviewers introduced themselves and the study and noted observations in addition to conducting the interview which typically lasted half an hour to an hour. Debriefs took place each evening within the research team to share insights and discuss areas needing further probing [35]. Interviewing stopped when saturation was reached [36]. Interviews were transcribed and simultaneously translated by the research team and two additional transcribers; a quality-check was performed on 20 transcripts against the Chichewa audio. All English transcripts were checked against the English audio by the lead author who also checked final transcripts, which were coded and analyzed in NVivo Pro 12.

Codes were generated deductively, based on the interview guide, and inductively through open coding; in-vivo coding i.e., coding according to expressions by respondents such as “drugs are fierce”, was also used. A code book was developed, discussed, and expanded as the analysis evolved. A second researcher coded 10% of randomly selected interviews showing the same understanding of the data. Several coding rounds and charting tables ensured nuances were captured. Drawing on thematic analysis [37] pertinent themes were identified in relation to men’s engagement with HIV services and ART using the socio-ecological model.

## Results

We present findings on community perceptions of HIV, ART and PLHIV followed by the various forms of stigma experienced or perceived by MSW interviewed. Masculinity issues pervaded MSWs’ personal accounts as cross-cutting themes.

### Participant characteristics

Our study is based on 72 in-depth interviews with 39 MSW on ART at UFC (referred to as male ART clients); 17 MSW in communities in the district of Blantyre (named male community respondents) and 16 district stakeholders^2^, the only group that included women (Table 1). The median age of male community respondents and male ART client respondents was 9-10 years lower than that of stakeholders with MSW with suppressed VLs showing the greatest age range. The majority of MSW had secondary-level education whereas most stakeholders were educated at tertiary level. Half of the male ART client respondents with a suppressed viral load (VL) mentioned a partner compared to 70% of male ART client respondents with unsuppressed VLs. From the interviews with male ART clients it emerged that most (22 out of 39), irrespective of VL and age, had been on ART for more than seven years.

**Table 1:** Respondent Table

Respondent Groups	Male (n = 68)	Female (n = 4)	Median Age [age range]	Completed Secondary Education	Living with partner
**Male ART clients -suppressed VL**	16		35 [18–54]	n = 10	n = 5
**Male ART clients - unsuppressed VL**	23		34 [18–53]	n = 13	n = 13
**Male community respondents**^a^	17		35 [24–51]	n = 9	n = 11
**Stakeholders**	12	4	44 [26–61]	n = 16	n/a
^a^It emerged during the interviews that four male community respondents were HIV−positive and on ART at local health facilities					

### HIV and ART perceptions projected onto PLHIV

Dominant views of HIV and ART in the communities varied greatly – particularly between stakeholders and the other respondent groups: stakeholders painted an overall positive picture of PLHIV living longer, healthier lives, better tolerated ARV regimens and a drastic reduction in stigma. A greater number of participants, irrespective of age and HIV- or VL-status, depicted negative community perceptions with HIV being seen as “the end of one’s life” (male community respondent, 25 years) or associated with a philandering lifestyle. Respondents portrayed communities as excluding or ignoring PLHIV in social activities, not wanting to associate with PLHIV, mocking or gossiping about them, viewing them as “non-productive” and “not fit enough to be part of the society”, and described stigma by association and direct discrimination.

When you have gone for drinking, you cannot buy your drink and join those that know you to have HIV as these people get to think that if I stay with this HIV person what will people think about me? Even when playing football, people say that “he does not have energy, that one, do not touch him, he will fall down”. (male community respondent, 26 years).

While community respondents viewed ART as helpful and prolonging lives, many perceived others in the community to hold negative views. “Some say the drugs are dangerous and others say they are deadly. The drugs are not deadly drugs, but they prolong the life of a person who has been found HIV-positive as long as they comply with the prescriptions” (male community respondent, 44 years). A salient community concern was having to swallow (large) pills daily, pills described as “deadly”, “bad”, “fierce”, or “shameful”. Some MSW also linked negative community perceptions of ART to visible side-effects: “their face gets to change and they start looking like a monkey” (male community respondent, 24 years), with some men on ART maintaining that this perception has stayed in people’s minds: “These people still think that even the ARVs that people are receiving now do have such signs and symptoms that one can be seen with” (male ART client, unsuppressed VL, 18 years). Despite lingering stigma, some participants viewed communities to be in transition. “It’s unfortunate - we still have some people who feel that HIV is for people who are promiscuous but the majority, they have come to realize that it is an infection or that anybody can contract it” (female stakeholder, academic, 1).

#### Stigma intertwined with masculinity considerations

Many male clients on ART experienced pill-taking as easier than anticipated with fewer or temporary side-effects. A few male clients around 40 years and above struggled with longer-lasting and visible side-effects such as veins protruding, sores or numbness in leg which made them recognizable as PLHIV. “I developed some body sores and these also left marks on my legs” (male ART client, unsuppressed VL, 50 years). The potential effects of ART on sexual performance, libido and fertility were a concern among younger and middle-aged men; for a young man, poor sexual performance led to interrupting ART occasionally.

R: When you compare your performance before starting ARVs and after taking ARVs there is really a disturbance and reduction in performance… When you go out with a woman you only realize you have only been able to only have sex with her in one round, it really disappoints because the woman thinks you are a weakling, yet the ARVs are the ones making you weak.

I: Have you yourself ever stopped taking ARVs at some point in time because of the same issue?

R: Yes, I have…It happens that when I have done only one round, I sleep the whole night and I do not even desire to still do more sex. Things like these are really challenges.

I: What year was it when you had opted to skip ARVs because of this?

R: It is generally all the years but now I just came to accept it that this is how things are like now. (male ART client, unsuppressed VL, 29 years)

Masculinity considerations played an important role for men in their health-seeking behavior. Stakeholders portrayed men as unlikely to live openly with HIV and commented on men’s dislike of doctors, going to hospital as a last resort, preferring not to be seen at health facilities and favoring private facilities. Some male stakeholders highlighted masculine notions of not wanting to display a problem publicly or ask for help, especially if they held higher social positions.

But somebody who has got a decent job, say for example, like myself, if I was HIV positive for instance, chances are, I would not declare my status…. if I have got a problem I would go, maybe like to a private hospital for instance, I would pay for services so I do not need to say it in the open because to them, somebody who comes out in the open, it’s like you are asking for help. (male stakeholder, Academia, 2)

Men living openly with HIV were seen to display a different type of masculinity which showed little interest in meeting societal expectations.

They are still men, they still maintain some of the characteristics that men are, but their focus in relation to HIV is totally different, it’s still being on treatment and having better health and helping someone out and they don’t care what people say about it. (male stakeholder, NGO, 3)

#### Men viewed differently throughout the life-course

Men’s age affected stigma levels and differentially affected testing uptake for male community respondents with younger men making more use of testing services despite exposure to higher stigma. While most male ART clients, irrespective of age, described how they tested when they were sick, most male community respondents spoke about wanting to know their HIV-status, some through mobile testing services. Community respondents below the age of 35 talked about testing regularly, with the wife, because the parents were positive or for a medical report; only one depicted testing because of feeling sick. One young man from a community had self-tested, a method many preferred, even if not widely available. Despite evidence to the contrary, younger men were perceived by all respondent groups to be reluctant to test for fear of stigma, isolation from friends or the thwarting of future plans. “It’s mostly young ones that are afraid to be known and being seen by friends. In fact, they are shy… VCT, it’s not an easy process” (male ART client, unsuppressed VL, 26 years).

**Fig. 1:**
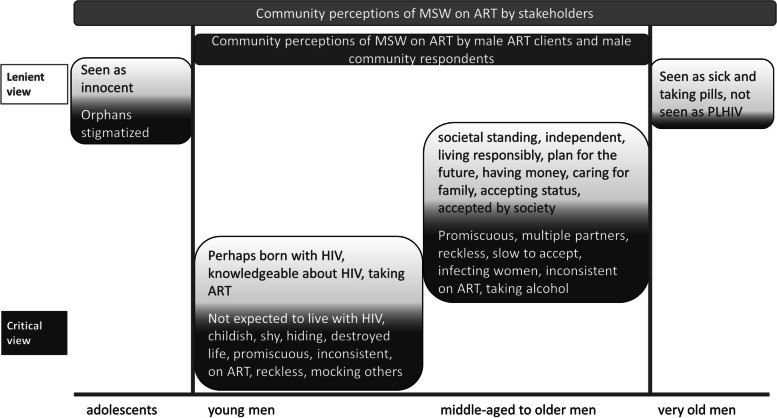
Age-related community perceptions of MSW on ART by respondent groups. Legend: Lenient community views are shown on a light background, critical community views on a dark background

Respondents saw age as an important factor in the community perception of MSW living with HIV (see Fig. 1). They described older men as more responsible, accepting their status, committed to taking ART and caring for their families. Middle aged and older MSW already had a standing in society, had responsibilities, could show achievements in having many children and an income. As a result, they were not exclusively defined by their HIV status. By contrast, younger men were seen as living “recklessly”, having a problem with their HIV-status, afraid to be ridiculed, to lose or not find a partner and shunning or stopping treatment.

There is a big difference: older people are mature, so they understand issues better than young people and they are at least committed to ART than younger men. Most young men shun treatment; they are shy, afraid of friends and above all young people lack responsibility. (male ART client, suppressed VL, 45 years)

Sexually active young MSW were thought to be blamed for their infection, whereas older men’s risk behavior, despite also being seen as “reckless”, would be tolerated.

In our culture, if a man is involved in risky behaviors, it’s a little bit acceptable… for a man it is perceived normal…for younger men, it’s like, no, these people are involved in promiscuous behaviors, that’s why they have HIV” (male stakeholder, NGO, 2).

The difference seemed to be the cultural acceptance and condonement of older MSW’s behavior: “They say, men are allowed to be polygamist, they are allowed to have multiple wives, multiple girlfriends” (female stakeholder, Health Profession, 8). MSW in their middle years would not be solely defined by their health or HIV-status, “maybe the health is not everything, it is the money” (male stakeholder, NGO, 1). Also, they were viewed as possibly infected by caring for others.

The community is more of accepting the older men who are living with HIV than the younger ones. The younger ones it’s like the perception is you have already destroyed your life before you have started it. You were promiscuous… For older men, it’s like the situation is accepted to a certain extent…You have lived part of your life, so you can manage the remainder. (male stakeholder, NGO, 3)

Extremes of age were regarded differently. In stakeholders’ community perceptions, adolescents were seen as infected through mother-to-child transmission and regarded as innocent. The adolescents themselves however, anticipated similar stigma levels, despite a different transmission route. Stakeholders also distinguished very old men from older MSW on ART.

I would say it’s very rare for older men, it’s very rare to classify them. Most maybe we say even, if he is sick, no one knows he is taking medication maybe because of the old age (male stakeholder, Church, 1).

Figure 1 contrasts community perceptions by MSW on ART with those of stakeholders. Male community respondents largely held the same views as male ART clients with both groups contrasting younger and older men. Stakeholders held more nuanced community views of MSW stretching from very young to very old men.

### From outside to inside perspective: men’s fears and experience of stigma

#### “My relatives know…but my friends don’t” - men anticipating stigma from outsiders

Anticipated stigma played a major role for MSW in how they might be perceived. “I would be afraid as to how my immediate relations would treat me like but at the same time I would also be afraid as to how the society in general would treat me like” (male community respondent, 31 years). While the vast majority of MSW on ART spoke about disclosing their HIV status to relatives and their partner, they were selective in who they trusted yet we noticed no difference in disclosure behavior by VL status. Most older MSW explained their status to the family, some as a means of receiving help in future or as an explanation for their poor health. “I told my wife, but I also told all my relatives like my sister and my younger brother because they were the ones who were seeing that I was deteriorating.” (male ART client, unsuppressed VL, 37 years). Some middle-aged men with suppressed and unsuppressed VLs disclosed to work colleagues, friends or neighbors to motivate them to test. Others restricted disclosure to their partner and planned their life so nobody would realize they were taking ART.

Even here no one knows that we are on ART; it is only my wife who knows about our status…Even at work, no one was told about my status, and the fact that most of the time, I do come here [to the health facility] on appointment basis, I have always made sure that I plan in time so that it would not collide with my work schedule. (male ART client, suppressed VL, 39)

A few MSW of different ages and VL-status did not share their status with family members, “I never shared this to anybody; I did not even inform that brother of mine who took me to the hospital. I kept it to myself” (male ART client, unsuppressed VL, 45 years). The same man revealed, however, that he eventually informed his wife “because she was close to me and it could have been hard for me to take medication in her presence without her knowing” (idem). One young man, born with HIV, described his elaborate efforts to conceal his HIV-status from his uncle’s family.

I stay with my uncle but have never told him that I am HIV-positive. This is because when I sat down and thought about it, I know, that if I tell him there will be discrimination against me and other things. Most people who talk about HIV say that others don’t relate with them well because of discrimination…. We sleep 6 boys in the boy’s quarter, and my uncle sleeps in the main house. So, what happens is that I wake up at 2am and start reading while others are still sleeping so I wake them up at 4 o’clock to get ready and draw water. So when I am going to take a bath is when I get my drugs. I put them in a plastic bag together with a flexa foam [sponge] because they make a noise. Then I put it in my hand like I am washing my face and drinking water. (male ART client, suppressed VL, 18 years)

Some MSW born with HIV did not disclose their status but consented to their caregiver or guardian (mother, aunt, uncle, in-laws, brother, sister) to inform relatives, yet drew a line with the mother sharing the result within the family. While friends played an important role, particularly for young men, telling a friend about one’s HIV infection could signal the end of the friendship.

Fear is there because you think of what your friend’s reaction will be unlike disclosing it to your relatives who understand because you’re a relative but for people you are not related to, they can just abandon you. (male ART client, unsuppressed VL, 18 years)

Knowing who to trust was difficult. Some younger and older MSW excluded friends categorically. “I have not disclosed to my friends because I know that when I do, I will be discriminated” (man in community, 51 years). The fear of a break-up of the relationship was a further barrier. “So, during courtship I did not disclose to her that I have the virus, because I loved her so much…. so, I explained to her when she was already pregnant” (male ART client, unsuppressed VL, 39 years).

Another fear for MSW to be recognized as PLHIV was expressed in having to carry rattling ARVs. One man recounted how he used his laptop bag to carry the bottles of ARVs home to stifle the sound. Another talked of his discomfort of travelling: “This is when I miss taking my medication…because I do not like how they sound when carried in a bottle” (male ART client, unsuppressed VL, 29 years). The general preference to be inconspicuous as MSW on ART was extended to facility visits. Some MSW decided to obtain treatment at faraway clinics where nobody would know them only to discover their neighbors had the same idea.

There’s a clinic in x but I am used to coming here and also for privacy, I don’t want to be seen by people from where I am staying especially my neighbors, but sometimes we still meet neighbors here and then you tend to wonder, “that so, neighbor is here as well”. (male ART client, suppressed VL, 39 years)

Being seen at the clinic by friends or neighbors and talked about was a major concern expressed by younger and older MSW on ART irrespective of VL status. This was also reflected in seeing a man lingering around the entrance of the facility until the person he knew in the waiting area had disappeared. Only then did he enter the facility. This observation further underscored MSW’s distrust of others to maintain discretion: “People do not keep each other’s secret as much as we both have come here as others will still talk after leaving this place to say that so and so has the virus” (male ART client, suppressed VL, 26 years). In addition, some MSW did not trust young health staff to keep confidentiality, others, mainly younger men, did not want to mix with women at the facility for fear that women may gossip about them; young MSW were also afraid of what older MSW would think about them and of having to expose their sexual life at the clinic.

They also ask you that why have you made the decision to come and test for HIV…and maybe you have really slept with a girl, and this girl is the daughter of the woman asking you, honestly to us as younger men it makes us uncomfortable because it is our private life. (male community respondent, 24 years)

A strategy not to meet people who could spread rumors was to arrive early or late at the facility.

I think the reason for coming in the evening is hiding, they don’t want other people to know that they are on treatment. If you are suffering from malaria, don’t you come openly? They go to the hospital openly saying I am suffering from malaria. But this HIV, everyone just thinks that they committed a crime, they trampled on thorns, that is what makes people to be coming here in the evening and if you would have been opening at 4 am, you would have seen people coming in larger numbers. (male ART client, unsuppressed VL, 46 years)

MSW of all ages anticipated stigma in meeting others at health facilities, yet none of those interviewed described experiencing stigma from health personnel for being HIV-positive. They spoke of friendly staff, being treated respectfully, not being condemned, and receiving helpful counseling and advice.

Some MSW gradually overcame anticipated stigma. A mechanic first hid his ARVs at work and then decided “if I get busy with what people talk about, I will not do anything in my life” (male ART client, unsuppressed VL, 32 years). A young man who had to work late one night and could not take the ARVs on time, started taking the pills at work in front of others, “if they laugh at me, I don’t care because next time it is them who can also get it” (male ART client, unsuppressed VL, 24 years).

#### Experiencing stigma and MSW’s coping mechanisms

The most common form of stigma experienced by MSW was being talked about, insulted or ridiculed. “Some people mock me after realizing that I am on ARVs. Either they suspect that I am on ARVs or they know that I do take ARVs. Sometimes they just want to embarrass me in front of other people. (male community respondent, 44 years). MSW on ART depicted feeling hurt, leaving the group, joining in the conversation to rectify information or ignoring comments by others. Enacted stigma in terms of being treated badly was narrated mainly by men above the age of 30 and related to being left by the partner when diagnosed with HIV, being told off by a relative for taking ARVs in the household or living in a hostile neighborhood:

In XX (town) I had problems with the people I was living with close by. They hated me and discriminated me to the extent that I lost hope of life; to them I was as good as a dead person. This prompted me to ask for a transfer from XXX clinic to here. The problem I had was nothing to do with the hospital but where I was staying in XXX. (male ART client, unsuppressed VL, 53 years)

It could also express itself in workplace discrimination:

…for me to be fired at X was because I was found HIV-positive… When I got sick, my boss asked me to tell him what it was, so I did. So, he said I don’t want to work with people who are sick, you have to go. (male ART client, unsuppressed VL, 38 years)

Discrimination was, however, not as prominent in men’s narratives as receiving stigmatizing comments.

#### Internalized stigma

Self-stigma emerged as a minor theme in male ART clients’ narratives. The statement of a young man blaming himself for his HIV infection was one of few statements that showed internalized stigma: “looking at my behavior, I did deserve to be found with HIV” (male ART client, unsuppressed VL, 24 years). Young men felt under pressure to conform to masculine norms of showing virility – “the friends we chat with are also the ones who encourage us that if a guy is just staying and does not even have a girlfriend, that person is not a man” (male community respondent, 27 years). Despite these expectations, some young MSW withdrew from friends or decided not to engage with potential sexual partners for fear of passing on the virus. Conversely, a slightly older man refused to self-stigmatize.

When I look at someone who is negative and another one who is positive, from my point of view, I regard myself as similar to the one who is negative. I don’t regard myself in any way just because I am taking this medicine. I don’t discriminate myself, by looking at others as normal and that I am not, no. I associate freely with everyone. (male ART client, unsuppressed VL, 39 years)

#### Older men’s resilience to stigma and wider masculinity repertoire

Men above 40 years, regardless of VL status showed more resilience to stigma and reflected a greater range of masculinities than their younger counterparts: caring for the well-being of the family and providing for their future, showing responsibility, having one’s social standing recognized by society were additional sought-after masculine expressions and gave older men more freedom to ignore societal masculinity expectations towards them. They mentioned no need to prove their virility: “if you are a married man, you don’t need to have a girlfriend” (man with unsuppressed VL, 42 years), spoke confidently about not “sleeping around” and told people off who tried to stigmatize them:

Most people insult you… As someone who is taking medication, those things don’t bother me… I tell them that it is not everyone who has contracted the disease because of sleeping around with women. Sometimes you just discover you are HIV-positive and you wonder how you got it, because you know you don’t sleep around with women. (male ART client, suppressed VL, 55 years)

They did not mind being recognized as a PLHIV when testing or going for treatment – “I even carry my bottle of medicine in my pocket whether it will make a sound or not, I do not mind” (male ART client, unsuppressed VL, 47 years). Their own well-being and that of the family mattered more than other people’s opinions which they perceived as a big difference to younger men.

Men like me do not care either; they will be discriminated against or not because we are able to make decisions on our own unlike youth, they make decision based on the influence of friends. (male ART client, unsuppressed VL, 53 years)

Older age gave them confidence they may not have had as younger men:

These youths might be feeling shy when it comes to testing in the communities like here because their fears will be such, such people will see me there. Yes, but for us, the older men we know that it is better to come out openly because maybe our time has gone, we only remain with few days…There is no reason to hide, I have to be getting treatment whether it’s X (facility), whether my children know about it or not, what I know is that I have saved my life. (male community respondent, 48 years)

Yet, some young men also refused to be stigmatized – “ARVs are not drugs one can be ashamed of” (male ART client, unsuppressed VL, 19 years). The mode of transmission made some younger men more resilient: “I don’t really care when people talk about it because this is something that I was born with” (male ART client, suppressed VL, 29 years). Older age alone was therefore not the only factor for acting independently of society’s expectations.

**Fig. 2:**
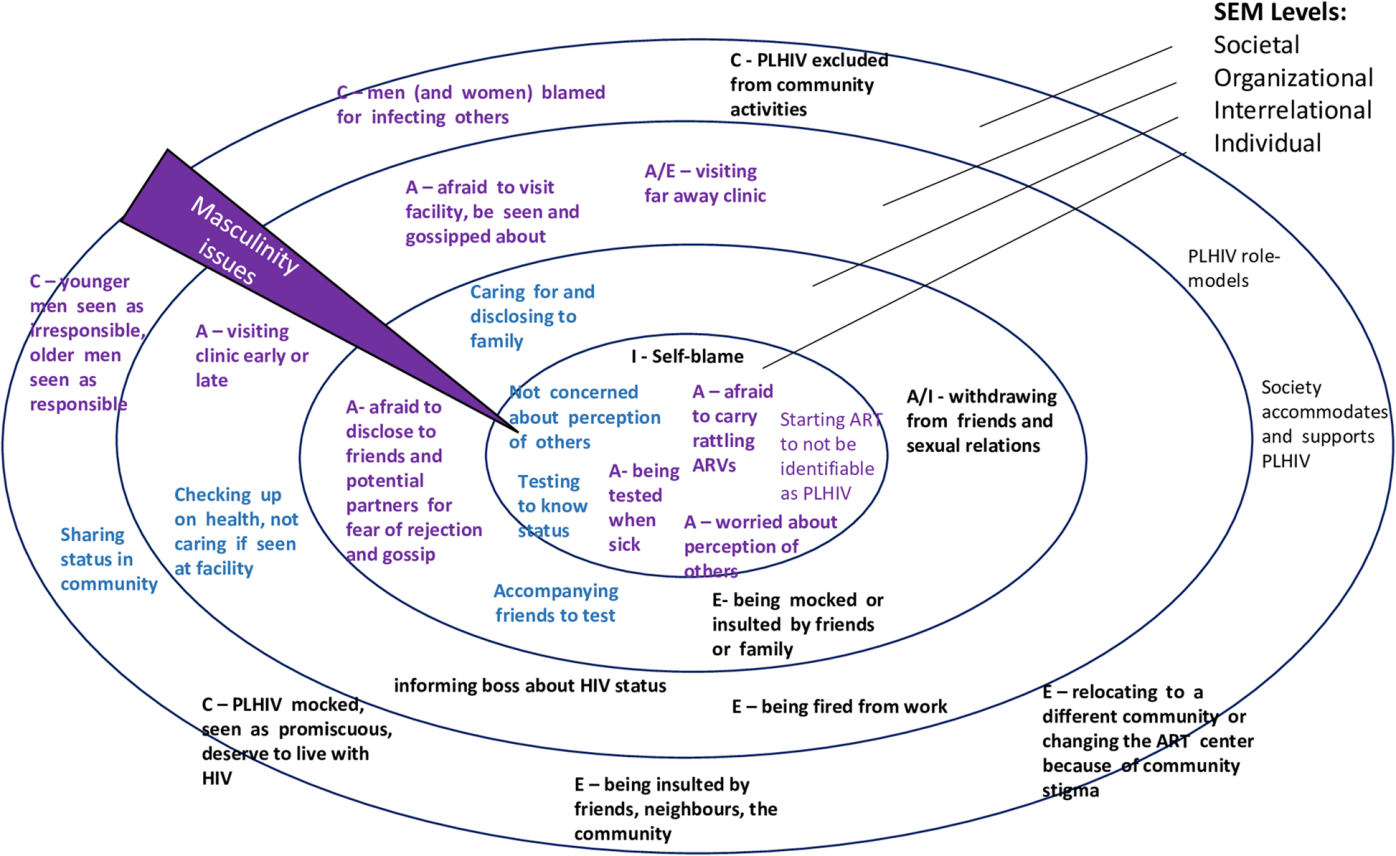
Stigma, masculinity and HIV at different levels of the socio-ecologic framework. Legend: A: anticipated, C: community-level stigma, E: enacted stigma, I: internalized stigma. Bold: mentioned by MSW on ART, not bold: mentioned by stakeholders, purple: linked to masculinity notions, blue: transformative masculinities

Figure 2 summarizes the stigma mentioned by respondent groups and MSW’s experiences and behavior at different societal levels with masculinity issues cutting across all levels.

## Discussion

Our study has shown how lingering negative community perceptions of HIV and ART coupled with masculinity considerations had repercussions for male PLIHV as individuals, their relationships, facility visits and interactions in the community. As Fig. 2 illustrates, some MSW subscribed to hegemonic masculinity ideals such as wanting to appear strong and independent, not testing for HIV when one feels healthy, afraid to lose one’s reputation; others confidently expressed and lived by other masculinity ideals, e.g., taking responsibility for one’s life and that of family and friends independent of other people’s opinions and did not feel compelled to meet societal expectations towards them. This was more pronounced in older MSW, who were able to draw on a greater spectrum of masculinities than younger men and highlighted the importance of the life-course and age-dimension of stigma and masculinity expressions for MSW in Blantyre.

### MSWs’ life-course and notions of masculinity intersecting with stigma

We view the “life-course” as a useful concept for studies on men and HIV both for changes within men’s own life-course (before and after taking ART) as well as age-related changes in men’s sexual life and standing in society. The life-course has been applied to HIV prevention in terms of vulnerabilities of men and women [38] and HIV testing [39]. We concur with Johnson and colleagues [39] that aging is primarily studied in the context of epidemiological HIV trends [40, 41], and that applying a gendered life-course approach would enhance HIV literature. Yet, beyond HIV-testing we propose to analyze “constructions of “respectable” midlife-older age” [39] vis-à-vis younger age to examine how the life-course affects men’s experience of stigma, uptake and adherence to ART in a quest for more targeted services for men.

Virility and sexuality were interpreted differently according to age and HIV-status. Virility was upheld for all men with the expectation that particularly young men should prove their manliness in being sexually active. Paradoxically, if their virility led to an HIV-infection, they became stigmatized. Young men who interrupted ART to have the stamina “for more rounds” also paid for their behavior with higher viral loads. Concerns about the health implications of normative masculine behavior have been raised in many studies and reviews over the last 20 years [42–46] with Hunter [47] pointing out fatal consequences of living up to societal masculine ideals in the early HIV epidemic in South Africa without the provision of ART. He traced “isoka” - the Zulu notion of having multiple partners - from a concept for both gender groups with reproach levied against those overstepping the mark to the concept becoming restricted to young men with no imposed sanctions for having too many concurrent partners. Hunter further pointed out that the custom is becoming problematized in the era of HIV. Hunter showed how societal gender and age expectations evolve with socio-economic and political changes and impact on societal views of acceptable and unacceptable behavior. Age-related masculinity and stigma notions thus evolve over time.

Our study showed more tolerance towards promiscuous middle-aged and older MSW with societal standing. This may have provided older men the freedom to be less concerned about stigma, to discard societal expectations (e.g. the need to have a girlfriend alongside the wife) or to elevate one masculine ideal (social standing) against another (virility). In contrast to our findings, studies in Uganda noted stigma to be high toward older men [48] and access of services dependent on men’s maneuverings of notions of respectability (fidelity in marriage, children, role of provider) versus reputation (defined by men as showing virility, strength and expending time and money for socializing) [49].

Our finding that stigma in its different forms and manifestations among men living with HIV in Blantyre is closely intertwined with notions of masculinity aligns with other studies [24, 26]. Wyrod highlighted HIV-positive men’s experience of conflict between the masculine ideal of the breadwinner and their unemployment and poverty situation [50]. In Blantyre we identify social standing, sexuality and health as dominant hegemonic masculine ideals [27, 28].

For MSW in communities (unlike MSW on ART) the narrative of knowing one’s status for staying healthy superseded testing and masculinity barriers and was facilitated by the convenience of mobile and self-testing. For older MSW on ART, the importance of their own life and health and their family’s well-being and future outweighed meeting societal expectations in terms of virility or concealing one’s HIV-status, yet some young and middle-aged men also took ARVs in front of others. We are hesitant to call these “subordinate” or “alternative” masculinities in the logic of Connell’s model as these men confidently displayed their behavior independent of societal normative expectations thus adding different masculine ideals and perhaps slowly changing these normative ideals as depicted in the context of fatherhood in South Africa [52]. We therefore prefer the term “transformative” masculinities, denoting an extension to masculinities in flux. Re-defining masculinity ideals due to living with HIV and gaining the respect of other men has been reported in other sub-Saharan studies [53, 54] and could become a catalyst for transformation. Older age might be an important but not the sole contributing factor for this transformation. More research is needed to explore an enabling environment for men to cultivate transformative masculinities.

### Stigma – a barrier for adherence and retention?

In line with other studies [55, 56], we found that community perceptions of PLHIV were internalized by PLHIV and led to stigma in its various forms and at all levels of SEM, see Fig. 2. In our study, negative community perceptions towards ART created an additional hurdle for men to engage with ART. For men on ART, experienced and anticipated stigma were also expressed by MSW with suppressed VLs thus highlighting that stigma does not need to be a barrier to ART adherence and retention. This finding would need further exploration in other contexts as recent reviews of qualitative studies underscore stigma as a barrier to ART adherence and retention [51]. Quantitative studies found stigma to be “associated with lower medication adherence” [56, 57]. Mixed methods studies may provide new insights into the significance of stigma for men’s adherence and retention.

### Negative perceptions of HIV continue to be fueled by the “us” and “them” dichotomy

Herek argued in the 1990 s that AIDS-related stigma^3^ was linked to being viewed as “responsible” for contracting a “fatal”, “contagious” and disfiguring disease “regardless of its specific epidemiology and history” [58]. We found negative images of HIV, ART and men living with HIV persisting despite decades of community awareness, a national ART roll-out and virtually no transmission among those with suppressed viral load [59]. Ignorance of ART has been linked to stigma towards PLHIV [60], yet in our study, widespread misconceptions and negative community attitudes remained despite familiarity with ART indicating that ART provision alone may not be sufficient to overcoming stigma [50, 61]. There was, however, limited knowledge of the fact that a suppressed viral load prevents onward HIV transmission (meaning individuals are no longer infectious or “contagious”) a factor that could contribute to adherence at individual level and reduce fear of contagion at community level.

Stigma tends to be directed to people who are perceived different in some form or other [62]. Community views in our study showed highly stigmatizing attitudes towards ART and (male) PLHIV. Yet MSW on ART who experienced the benefits of treatment in terms of restored health, being able to work again and becoming indistinguishable from other MSWs came to hold markedly different views. Other studies have found that those not initiated on ART perceived higher barriers than those already on treatment [63], calling for more involvement of PLHIV in HIV sensitization programs.

While our findings show that MSW on ART were acutely aware of community perceptions and behavior towards them as PLHIV in terms of being (potentially) mocked or excluded, their reactions at the interpersonal level in terms of disclosure and non-disclosure was based on a dichotomy of trust, a variation of the “us” and “them”: blood relations, wives or long-standing partners and the extended family were regarded as trustworthy and loyal. Outsiders such as friends or potential partners would have to be screened carefully. MSW’s in-group preference for disclosure (partner and family) has also been noted in East and South African ART studies [64–66]. Overcoming the divide between “us” and “them” would call for building a bridge from both ends: the community showing greater understanding of ART, openness and acceptance of PLHIV and male PLHIV of different ages building up trust beyond their partners, family and selected friends. This would be a giant step towards overcoming stigma in line with Gilmore and Somervile’s slogan: “we are all us” [67].

### Limitations

The findings of this study are based on the views of stakeholders, men on ART and men in the communities triangulated to deduce community perceptions about ART. While this has proved useful in highlighting differing perceptions, we believe an extended period of living in one of the communities would have added more observational data and insight on how younger and older male PLHIV are treated and how they engage with their communities. We recommend including more participant observation in future research on stigma.

## Conclusions

HIV-related stigma, as we have shown, intertwines with masculinity and men’s life-course, and has not yet been overcome in Blantyre. Stigma manifests itself in community attitudes towards HIV and ART with older images of HIV and ART persisting and new knowledge about the non-infectiousness of suppressed PLHIV not being widely known. Attitudes and behavior towards PLHIV, anticipated stigma and self-stigma on the part of PLHIV were independent of viral load status. The intersectionality of stigma and masculinity notions throughout the life-course of MSW on ART but particularly for younger men calls for more discreet and male-led HIV services at facilities and in the communities, nuanced interventions for men of different ages, and more inclusion of male PLHIV in the design and implementation of interventions. This will require more research into HIV-related stigma experienced by younger men, into enablers for “transformative masculinities” and how best to overcome the “us” and “them” divide.

## Supplementary Information

**Additional file 1.**

## Data Availability

The datasets generated during the current study are not publicly available in order to protect the identity of interviewees. Pseudonymized transcripts can be made available from the corresponding author on reasonable request.

## Footnotes

1 The team successfully completed an on-line research ethics course by FHI, https://www.fhi360.org/sites/all/libraries/webpages/fhi-retc2/index.

2 For the composition of stakeholders, see 2 section.

3 His use of the term AIDS-related stigma should be understood in the context of (a) less of a distinction made between HIV and AIDS in early HIV publications and (b) in the 1990s not many people had access to life-prolonging ART so more PLHIV developed AIDS-defining symptoms making PLHIV more visible.
