# Supplementary material for: “Deadly”, “fierce”, “shameful”: notions of antiretroviral therapy, stigma and masculinities intersecting men’s life-course in Blantyre, Malawi
Source: BMC Public Health. 2021 Dec 11;21:2247. doi: 10.1186/s12889-021-12314-2 (PMC8665632; doi:10.1186/s12889-021-12314-2)
Supplement: Supplementary file 1 — Additional file 1. [file 12889_2021_12314_MOESM1_ESM.docx]

# **“Deadly”, “fierce”, “shameful”: Notions of antiretroviral therapy, stigma and masculinities intersecting men’s life-course in Blantyre, Malawi**

SUPPLEMENT 1: COREQ 32-­‐ITEM CHECKLIST

Tong A, Sainsbury P, Craig J. (2007) Consolidated criteria for reporting qualitative research (COREQ): a 32-­‐ item checklist for interviews and focus groups. International Journal for Quality in Healthcare: 19:349 – 357

| No. Item | Guide questions/description | Comments/  Reported on Page # |
| --- | --- | --- |
| Domain 1: Research team  and reﬂexivity |  |  |
| 1. Interviewer/facilitator | Which author/s conducted the  interview? | Lead author and 2nd author, page 5 |
| 2. Credentials | What were the researcher’s credentials? | Page 5 |
| 3. Occupation | What was their occupation at the time of  the study? | Page 5 (assistants were working as research assistants, one was a master’s student) |
| 4. Gender | Was the researcher male or female? | Main researcher female, study team mixed, page 5 |
| 5. Experience and training | What experience or training did the  researcher have? | Qualified anthropologist, page 5 |
| 6. Relationship with participants established | Was a relationship established prior to study commencement? | The facility was in close contact with clients, the DHO with communities and stakeholders were approached 2 months prior to the commencement of the study. |
| 7. Participant knowledge  of the interviewer | What did the participants know about  the researcher? | Page 5 |
| 8. Interviewer  characteristics | What characteristics were reported  about the interviewer/facilitator? | Page 5 |
| Domain 2: study design |  |  |
| 9. Methodological  orientation and Theory | What methodological orientation was  stated to underpin the study? | Pages 4-5 |
| 10. Sampling | How were participants selected? | Page 5 |
| 11. Method of approach | How were participants approached? | Page 5 |
| 12. Sample size | How many participants were in the  study? | Page 6-7 |
| 13. Non-­‐participation | How many people refused to participate  or dropped out? Reasons? | Nobody, page 5 |
| 14. Setting of data collection | Where was the data collected? | Page 5 |
| 15. Presence of non-­‐  participants | Was anyone else present besides the  participants and researchers? | In 2 stakeholder interviews was a colleague of the stakeholder present but quiet |
| 16. Description of sample | What are the important characteristics  of the sample? | Page 6-7, Table 1 |
| 17. Interview guide | Were questions, prompts, guides provided by the authors? | Page 5 |
| 18. Repeat interviews | Were repeat interviews carried out? | No |
| 19. Audio/visual recording | Did the research use audio or visual recording to collect the data? | Page 5 |
| 20. Field notes | Were ﬁeld notes made during and/or after the interview? | Page 5 |
| 21. Duration | What was the duration of the interviews | Page 5 |
| 22. Data saturation | Was data saturation discussed? | Page 5 |
| 23. Transcripts returned | Were transcripts returned to  participants for comment and/or correction? | Only in cases where a word could not be understood on the audio were stakeholders approached to render the correct sentence. |
| Domain 3: analysis and  ﬁndings |  |  |
| 24. Number of data coders | How many data coders coded the data? | 2, page 5 |
| 25. Description of the  coding tree | Did authors provide a description of the  coding tree? | Pages 5-6 |
| 26. Derivation of themes | Were themes identiﬁed in advance or  derived from the data? | Both, see page 5 |
| 27. Software | What software, if applicable, was used to manage the data? | NVivo Pro 12, page 5 |
| 28. Participant checking | Did participants provide feedback on  the ﬁndings? | Health personnel were informed about initial findings and were interested in how to improve service provision. |
| 29. Quotations presented | Were participant quotations presented to illustrate the themes/ﬁndings? Was  each quotation identiﬁed? | Yes, pages 7-16 |
| 30. Data and ﬁndings  consistent | Was there consistency between the data  presented and the ﬁndings? | Yes, pages 6-17 |
| 31. Clarity of major themes | Were major themes clearly presented in the ﬁndings? | Yes, pages 6-17 |
| 32. Clarity of minor  themes | Is there a description of diverse cases or  discussion of minor themes? | Yes, 7-16, see in particular figure 1 and page 14 |
